# Supplementary material for: 3D visualization of mitochondrial solid-phase calcium stores in whole cells
Source: eLife. 2017 Nov 6;6:e29929. doi: 10.7554/eLife.29929 (PMC5703638; doi:10.7554/eLife.29929)
Supplement: Transparent reporting form [file elife-29929-transrepform.docx]

***eLife’s* transparent reporting form**

We encourage authors to provide detailed information *within their submission* to facilitate the interpretation and replication of experiments. Authors can upload supporting documentation to indicate the use of appropriate reporting guidelines for health-related research (see [EQUATOR Network](http://www.equator-network.org/%20)), life science research (see the [BioSharing Information Resource](https://biosharing.org/)), or the [ARRIVE guidelines](http://www.plosbiology.org/article/info:doi/10.1371/journal.pbio.1000412) for reporting work involving animal research. Where applicable, authors should refer to any relevant reporting standards documents in this form.

If you have any questions, please consult our Journal Policies and/or contact us: [editorial@elifesciences.org](mailto:editorial@elifesciences.org).

**Sample-size estimation**

- You should state whether an appropriate sample size was computed when the study was being designed
- You should state the statistical method of sample size computation and any required assumptions
- If no explicit power analysis was used, you should describe how you decided what sample (replicate) size (number) to use

Please outline where this information can be found within the submission (e.g., sections or figure legends), or explain why this information doesn’t apply to your submission:

No sample-size estimation was performed. We imaged as many cell regions in as many cell types as was feasible over the course of 2.5 years given limits on investigator time and access to the electron microscope. The results represent an extensive survey and are presented qualitatively.

**Replicates**

- You should report how often each experiment was performed
- You should include a definition of biological versus technical replication
- The data obtained should be provided and sufficient information should be provided to indicate the number of independent biological and/or technical replicates
- If you encountered any outliers, you should describe how these were handled
- Criteria for exclusion/inclusion of data should be clearly stated
- High-throughput sequence data should be uploaded before submission, with a private link for reviewers provided (these are available from both GEO and ArrayExpress)

Please outline where this information can be found within the submission (e.g., sections or figure legends), or explain why this information doesn’t apply to your submission:

Biological replicates are cells grown on EM grids in separate cultures on different days. Technical replicates are cells grown on either the same or separate EM grids in the same culture at the same time. Each biological replicate reported below was typically analyzed by collecting 2 to 5 full tomograms, which are technical replicates.

Number of biological replicates:

Untreated WI-38 fibroblasts	17

JC-1 treated WI-38 fibroblasts	3

FCCP treated WI-38 fibroblasts	2

Doxorubicin treated WI-38 cells	2

Mouse embryonic fibroblasts	3

Human dermal endothelial cells	1

HeLa cells	1

MCF10A breast epithelial cells	1

One mitochondrion without granules in an untreated cell culture was encountered in MCF10A cells and is described in the text (Figure 5D). Fifteen out of the 30 mitochondria observed in near-confluent fibroblast cultures lacked granules. An example appears in the supplement to Figure 5.

**Statistical reporting**

- Statistical analysis methods should be described and justified
- Raw data should be presented in figures whenever informative to do so (typically when N per group is less than 10)
- For each experiment, you should identify the statistical tests used, exact values of N, definitions of center, methods of multiple test correction, and dispersion and precision measures (e.g., mean, median, SD, SEM, confidence intervals; and, for the major substantive results, a measure of effect size (e.g., Pearson's r, Cohen's d)
- Report exact p-values wherever possible alongside the summary statistics and 95% confidence intervals. These should be reported for all key questions and not only when the p-value is less than 0.05.

Please outline where this information can be found within the submission (e.g., sections or figure legends), or explain why this information doesn’t apply to your submission:

With the exception of the estimates of calcium/phosphate granule densities, data is presented qualitatively. The basis for the density calculations and all assumptions made are described in the methods section.

(For large datasets, or papers with a very large number of statistical tests, you may upload a single table file with tests, Ns, etc., with reference to sections in the manuscript.)

**Group allocation**

- Indicate how samples were allocated into experimental groups (in the case of clinical studies, please specify allocation to treatment method); if randomization was used, please also state if restricted randomization was applied
- Indicate if masking was used during group allocation, data collection and/or data analysis

Please outline where this information can be found within the submission (e.g., sections or figure legends), or explain why this information doesn’t apply to your submission:

Samples differed by cell types and were not assigned to groups.

In cases where treatments were applied to cells, control cells and cells for treatment were plated in parallel, and the treatment plate was selected randomly.

**Additional data files (“source data”)**

- We encourage you to upload relevant additional data files, such as numerical data that are represented as a graph in a figure, or as a summary table
- Where provided, these should be in the most useful format, and they can be uploaded as “Source data” files linked to a main figure or table
- Include model definition files including the full list of parameters used
- Include code used for data analysis (e.g., R, MatLab)
- Avoid stating that data files are “available upon request”

Please indicate the figures or tables for which source data files have been provided:
